# MOF-Derived CoNi Nanoalloy Particles Encapsulated in Nitrogen-Doped Carbon as Superdurable Bifunctional Oxygen Electrocatalyst

**DOI:** 10.3390/nano13040715

**Published:** 2023-02-13

**Authors:** Li Wang, Jiewen Liu, Chuanjin Tian, Wenyan Zhao, Pengzhang Li, Wen Liu, Liang Song, Yumin Liu, Chang-An Wang, Zhipeng Xie

**Affiliations:** 1 Institute of New Energy Materials and Devices, School of Materials Science and Engineering, Jingdezhen Ceramic University, Jingdezhen 333001, China; 2 Department of Materials Science and Chemical Engineering, Stony Brook University, Stony Brook, NY 11790, USA; 3 State Key Lab of New Ceramics and Fine Processing, School of Materials Science and Engineering, Tsinghua University, Beijing 100084, China

**Keywords:** CoNi alloy nanoparticles, ZIF-67, bifunctional electrocatalysts, nitrogen-doped carbon

## Abstract

Carbon-encapsulated transition metal catalysts have caught the interest of researchers in the oxygen reduction reaction (ORR) and the oxygen evolution reaction (OER) due to their distinctive architectures and highly tunable electronic structures. In this work, we synthesized N-doped carbon encapsulated with CoNi nanoalloy particles (CoNi@NC) as the electrocatalysts. The metal-organic skeleton ZIF-67 nanocubes were first synthesized, and then Ni^2+^ ions were inserted to generate CoNi-ZIF precursors by a simple ion-exchange route, which was followed by pyrolysis and with urea for the introduction of nitrogen (N) at a low temperature to synthesize CoNi@NC composites. The results reveal that ZIF-67 pyrolysis can dope more N atoms in the carbon skeleton and that the pyrolysis temperature influences the ORR and OER performances. The sample prepared by CoNi@NC pyrolysis at 650 °C has a high N content (9.70%) and a large specific surface area (167 m^2^ g^−1^), with a positive ORR onset potential (Eonset) of 0.89 V vs. RHE and half-wave potential (E_1/2_) of 0.81 V vs. RHE in 0.1 M KOH, and the overpotential of the OER measured in 1 M KOH was only 286 mV at 10 mA cm^−2^. The highly efficient bifunctional ORR/OER electrocatalysts synthesized by this method can offer some insights into the design and synthesis of complex metal-organic frameworks (MOFs) hybrid structures and their derivatives as functional materials in energy storage.

## 1. Introduction

Electrocatalytic reactions including ORR, OER, and HER play an important role in fuel cells, metal-air cells, and water electrolysis. It is generally known that Pt-based catalysts are the most active catalysts for ORR and HER, while Ir/Ru-based catalysts are the most active in OER [1,2]. Unfortunately, the high cost and scarce resources of these precious metals, as well as the poor performance of multifunctional catalysts, have greatly limited their commercialization. Therefore, there exists an urgent need to develop catalysts with low cost, high catalytic activity, and stability. Non-precious metal catalysts (NPMCs) are particularly attractive potential materials for replacing precious metal catalysts. Researchers have proposed a variety of NPMCs, including transition metal nanocrystals, transition metal compounds, and metal-free heteroatom-doped carbon compounds [3], such as transition metal sulfides [4], oxides [5], nitrides [6], nitrogen oxides [7], carbides [8], and phosphides [9], but the majority of them are limited by low electrical conductivity and poor electrocatalytic activity. Transition metal nanocrystals are currently considered promising non-precious metal catalysts, but non-precious metal catalysts have problems such as agglomeration and poor stability. As a result, researchers have introduced carbon-based materials to solve these issues.

Carbon-based materials have a wide range of sources, large specific surfaces, and good electrical conductivity, but their intrinsic activity is low, so heteroatomic doping is required to boost their catalytic activity. Carbon skeleton doped with heteroatoms (e.g., N, P, B, and S, etc.) can effectively modify the electronic structure and chemical state, which may lead to more catalytically active defects for improved electrocatalytic performance [10]. Carbon-based materials are commonly researched for ORR but seldom reported as bifunctional catalysts for OER, owing to their low OER activity and unavoidable carbon corrosion at high potentials [11]. These problems may result in catalyst agglomeration and the formation of carbonates covering the active sites on the surface, thus causing catalyst deactivation [12]. Recent theoretical calculations have confirmed that transition metal particles encapsulated in N-doped graphitic carbon can effectively anchor transition metals and inhibit metal agglomeration. The presence of partial N in the carbon layer involved the interaction between the support carbon layer and the inner metal, which affects the local electronic structure redistribution and improves the catalytic activity and structural stability of the metal catalyst, allowing it to approach the precious metal benchmark [13].

In recent years, carbon derived from metal-organic frameworks (MOFs) has developed as a new method for producing functional carbon [14]. MOFs have highly ordered porous structures constructed through metal nodes and organic linkers. After pyrolysis in an inert atmosphere, the organic linkers decompose into carbon species. During the electrochemistry process, the metal elements are converted into metal oxides or remain in metallic states according to the reduction potential, and the porous structures can be maintained to some extent [15,16]. As a subclass of MOFs, zeolitic imidazole frameworks (ZIF-67) are an excellent precursor for carbon nanocatalysts because of the presence of abundant carbon and nitrogen species and the metal nanoparticles in ZIF-67 can be used as catalysts for CNT growth [17]. Although direct pyrolysis of ZIF-67 metal precursors can also yield highly graphitized N-doped nanocarbons, the high degrees of graphitization usually lead to reduced N content and porosity, which hinders ionic substances transport and catalytic activity [18]. Thus, metal elements were introduced to adjust their structures, and it was found that catalysts with bimetallic or polymetallic active sites could improve the catalytic activity and selectivity of various chemical transformations.

When MOFs are used as precursors, the resulting carbon-based materials can be divided into metal-free carbon-based materials and transition metal-nitrogen-carbon. Metal-free carbon-based catalysts avoid the problem of particle agglomeration and exhibit better stability compared to metal-loaded catalysts. A series of metal-free catalysts were synthesized using Zn in ZIF-8 that evaporates at high temperatures, but the resulting micropores allow limited mass transfer to the active site, so a graded porous structure was considered to improve catalytic performance. Qian et al. used an exfoliation strategy to design an interconnected network of reticulated carbon and then carbonized NaCl/ZIF-8 composites. The carbonized molten salt contributed to the ZIF-8-derived carbon graphitization, followed by Zn removal to produce microporosity, which with the microporosity formed by evaporation constitutes a graded porous structure [19]. In order to solve the problem of unsatisfactory catalytic performance due to framework collapse and active site agglomeration by pyrolysis. Shang et al. restricted the morphology and content of ZIF-67 to protected polyhedra in SiO_2_, followed by acid etching to wash away the metal species to obtain ZIF-67-derived metal-free carbon materials [20]. Materials containing transition metals can be thermally reduced by carbon when treated at high temperatures to obtain transition metal-nitrogen-carbon materials. In 2016, Xia et al. calcined ZIF-67 under a reducing Ar/H_2_ mixed atmosphere to generate N-doped hollow structures, and this work inspired a number of studies on MOF-derived M-N-C composite bifunctional catalysts [21]. To avoid the problem of nanoparticle aggregation, researchers encapsulated catalytically active metal nanoparticles in carbon nanotubes. Aniruddha Kundu et al. physically mixed CoNi-MOF precursors with dicyandiamide DCDA to prepare nitrogen-doped carbon nanotube-encapsulated Co_0.25_Ni_0.75_ alloys using a one-step carbothermal reduction method where hollow spherical CoNi-MOF was reduced to the metallic state by dicyandiamide without the use of a gaseous reducing agent [22]. Arpan Samant et al. synthesized NiCo-N-TC catalysts using melamine, pyridine dicarboxylic acid and Co and Ni self-assembled with the alloy particles confined to the tip, and the tip carbon and alloy particles were removed by acid treatment to form NiCo-N-TC-H hollow nanostructures with open heads and closed tails structure, while the NiCo alloy particles are uniformly distributed on the TC, increasing the specific surface area [23].

**Scheme 1:**
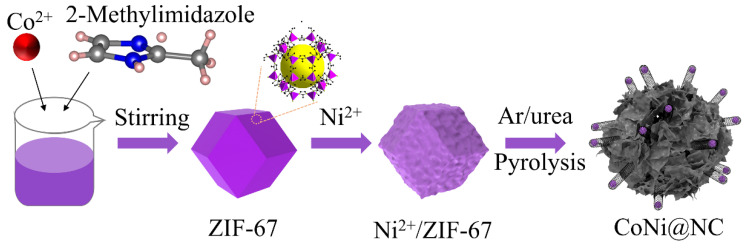
Schematic illustration of synthesis process of CoNi@NC.

As is shown in Scheme 1, we report the use of the organic linker of ZIF-67 as a carbon and nitrogen source, the decomposition of urea in the Ar atmosphere releases strong reducing gas NH_3_ to reduce Co and Ni species from CoNi-ZIF to form CoNi metal nanoparticles, which, in turn, catalyze ZIF-67 into N-doped carbon nanotubes. Furthermore, the decomposed NH_3_ can also provide an additional nitrogen source to improve the nitrogen doping level of carbon nanotubes. The internal CoNi alloy nanoparticles in this MOFs-derived nanostructure can provide a large number of active sites to catalyze the reaction effectively, while the carbon nanotubes act as a conductive network supporting the CoNi catalyst, which effectively prevents CoNi nanoparticles from aggregation and reduces corrosion [24]. In this work, we demonstrate that the annealing temperature is an essential parameter that can adjust the composition of CoNi@NC to optimize their electrocatalytic properties.

## 2. Experimental Section

### 2.1. Materials

Cobalt nitrate hexahydrate (Co(NO_3_)_2_·6H_2_O), nickel nitrate hexahydrate (Ni(NO_3_)_2_·6H_2_O), 2-methylimidazole, ethanol, and urea were all analytically pure and bought from Sinopharm Chemical Reagent Co., Ltd., (Shanghai, China).

### 2.2. Preparation of CoNi@NC-

10 g of 2-methylimidazole was dissolved in 200 mL of ethanol solvent and stirred to form solution A. Following that, dissolve 8.86 g Co(NO_3_)_2·_6H_2_O in the solvent and stir with 200 mL ethanol to make solvent B. Then the solutions A and B were mixed and stirred for 10 min to generate a purple solution and aged at room temperature for 20 h. The purple precipitate was centrifuged and washed three times with ethanol and dried in vacuum at 60 °C for 6 h to get ZIF-67. 1 g of ZIF-67 was put into 0.5 g of Ni(NO_3_)_2_·6H_2_O in 90 mL of ethanol solution under stirring for 6 h. The mixture was centrifuged and dried at 60 °C for 6 h to obtain Ni-doped ZIF-67 (Ni^2+^/ZIF-67). The resulting Ni^2+^/ZIF-67 and urea were placed in two separate porcelain boats in the furnace at a mass ratio of 1:10 (urea was placed at the upstream end) and annealed at 550, 650, 750, and 850 °C for 5 h under Ar atmosphere at heating rate of 3 °C min^−1^ to obtain black powders, which were noted as CoNi@NC-X (X represents 550, 650, 750 and 850).

## 3. Results and Discussion

**Figure 1:**
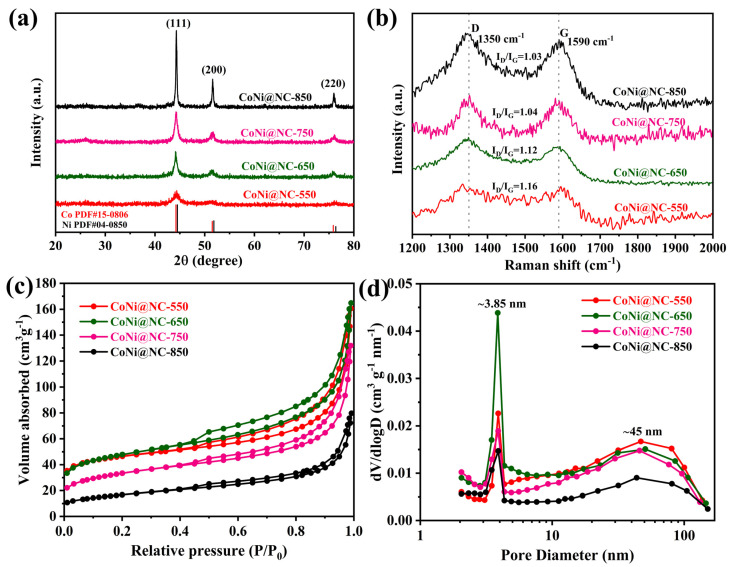
(**a**) XRD pattern of CoNi@NC. (**b**) Raman spectra. (**c**) N_2_ adsorption/desorption isotherms. (**d**) Pore size distributions.

The XRD measurement is applied to reveal the crystal structure of the obtained CoNi@NC synthesized at different temperatures. Figure S1 is the XRD of Co-ZIF and CoNi-ZIF. As shown in Figure 1a, typical peaks at 44.2°, 51.5°, and 75.8°, correspond to the (111), (200), and (220) crystal planes of the CoNi alloy, respectively. It is evident that the peak intensity increases with the increase of the pyrolysis temperature, indicating increasing crystallinity of the samples with the temperature increase. Meanwhile, the particle size of CoNi@NC intermetallic nanoparticles increases significantly, and the grain sizes of CoNi@NC-550, CoNi@NC-650, CoNi@NC-750, CoNi@NC-850 were calculated by Scherrer formula, which were 3.63 nm, 8.38 nm, 9.16 nm, and 25.35 nm, respectively.

Raman spectra were used to detect the degree of graphitization and defects of the CoNi@NC catalysts synthesized at different temperatures. The separate D and G bands are shown in Figure 1b at 1350 cm^−1^ and 1590 cm^−1^. The presence of the G band indicates that the catalyst is dominated by highly graphitized carbon following high-temperature pyrolysis, while the D band is caused by the insertion of defects in the carbon layers by processes such as doping and heat treatment. The degree of the defect and graphitization can be shown by the I_D_/I_G_ value. The higher the ratio, the more defects the sample contains and the lower the degree of graphitization. According to Figure 1b, it can be found that these samples increase in graphitization and decrease in sample defects with increasing pyrolysis temperature. Related studies have shown that higher I_D_/I_G_ is associated with more defects, which would enhance O_2_ adsorption and favor ORR [25,26], and lower I_D_/I_G_ indicates a higher degree of graphitization with higher conductivity, which facilitates electron transfer and thus increases electrocatalytic activity. This means that balancing the graphitization degree and defects can optimize the electrical conductivity and increase the active sites [27].

In order to ascertain the specific surface area and pore size distributions of the CoNi@NC as it was synthesized, the N_2_ adsorption/desorption isotherms analysis was used. As illustrated in Figure 1c, CoNi@NC-650 exhibits a combination of type I and type IV isotherms, indicating the existence of the hierarchically porous structure. The rapid absorption of N_2_ at low temperatures indicates the presence of small center pores, which are mostly nanoparticles formed by ZIF-67 [28]. Furthermore, the hysteresis loop in the region (P/P0 > 0.45–1.0) indicates the presence of large center pore structures, which are mostly formed from nitrogen-doped carbon. The same is true for the other catalysts, so small center pores and large center pores coexist in all samples. The specific surface areas measured by the Brunauer-Emmentt-Teller (BET) method for CoNi@NC-X were 160, 167, 119, and 60 m^2^ g^−1^, respectively, and their corresponding pore volumes were 0.21, 0.23, 0.19, and 0.12 cm^3^ g^−1^. The decrease of BET-specific surface area with increasing pyrolysis temperature may be attributed to the aggregation of metal nanoparticles as well as the collapse of the pore structure [29,30]. The higher specific surface area and pore volume may lead to more exposure to the abundant active sites, which is beneficial to electrocatalytic performance for ORR and OER. The four samples’ pore sizes are depicted in Figure 1d, with all four samples exhibiting peaks of about 3.85 nm and 45 nm. The 45 nm peaks may be related to the production of gas and the breaking of certain polymer spheres during pyrolysis [31]. The small mesopore (~3.85 nm) reduces the transport resistance of the reactants to the ORR, while the large mesopore (~45 nm) stores electrolytes and speeds up the reaction rate. Therefore, the graded porous structure allows the catalyst to expose more catalytic active sites and reduces the diffusion resistance for promoting the transmission rate of oxygen species, which is helpful to increase the catalytic activities of the CoNi@NC catalysts [32,33].

**Figure 2:**
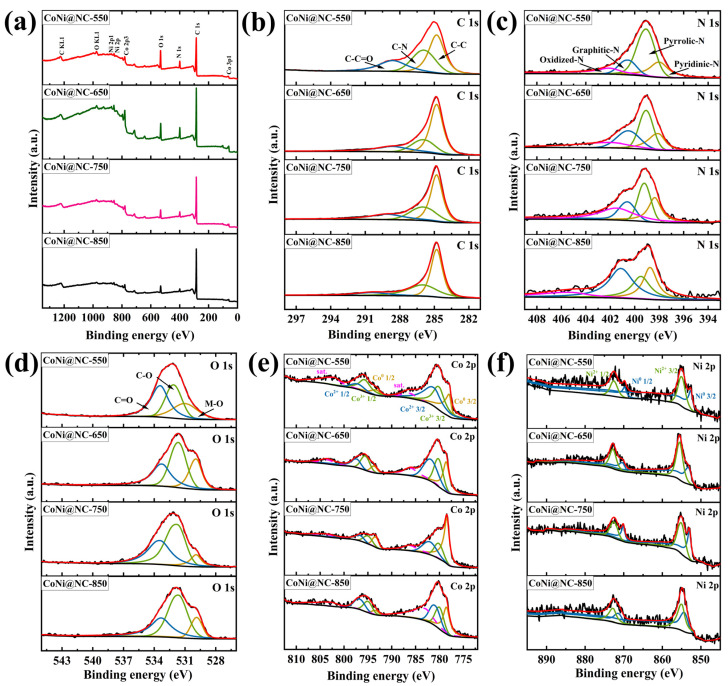
(**a**) Survey XPS spectra, high-resolution XPS of (**b**) C 1s, (**c**) N 1s, (**d**) O 1s, (**e**) Co 2p, and (**f**) Ni 2p for CoNi@NC.

The chemical composition of the sample surface measured by X-ray photoelectron spectroscopy (XPS) is depicted in Figure 2. There are apparent characteristic peaks of Co, Ni, C, N, and O in the total XPS spectrum of CoNi@NC, proving the existence of the above elements (Figure 2a). The content of each sample of CoNi@NC is shown in Table S1, and it should be noted that the metal content measured by XPS is very low. This may be due to the fact that XPS is more sensitive to the surface and has difficulty detecting the metal signal within the carbon [34]. The typical C 1s XPS spectrum can be deconvolved into three peaks of 284.8, 285.94, and 288.67 eV, corresponding to C-C, C-N, and C-C=O, respectively. Nitrogen is doped into the carbon molecular skeleton, as seen by the C-N peak (285.94 eV) in the C 1s XPS spectrum (Figure 2b). The nitrogen content of CoNi@NC-X was about 10 atom%, and N 1s XPS deconvolution of the catalysts showed peaks for four nitrogen species, including pyridinic-N (398.06 eV), pyrrolic-N (399.07 eV), graphitic-N (400.05 eV), and oxidized-N (401.86 eV) (Figure 2c). Pyridine-N can significantly modulate the electronic structure of the nitrogen-doped carbon, facilitating oxygen adsorption at the catalytic active site [35,36]. A higher degree of graphitic nitrogen can reduce the electron transfer resistance, so the combined impact of high-density pyridinic-N and graphitic-N with lower electron impedance is advantageous to improving ORR activity [37]. N content of each CoNi@NC is shown in Table S2. Deconvoluting high-resolution XPS of transition metal elements was used to examine the transition metal valence states in the catalysts. Co 2p high-resolution XPS can be divided into three pairs of 2p_3/2_-2p_1/2_ split peaks at 778.58 eV and 793.73 eV for the pair of Co^0^ peaks, 780.20 eV and 795.56 eV for the pair of Co^3+^ peaks, 781.83 eV and 797.39 eV for the pair of Co^2+^ peaks and 785.99 eV and 803.54 eV of the two satellite peaks (Figure 2e). The high-valence Co also demonstrates the presence of Co-N. Likewise, Ni 2p spectra can be separated into two pairs of 2p_3/2_-2p_1/2_ split peaks at 853.27 eV and 870.38 eV for the Ni^0^ peak and 855.53 eV and 872.79 eV for the Ni^2+^ peak, respectively (Figure 2f) [38]. The peaks corresponding to the zero valent transition metals prove that the catalyst contains the corresponding transition metal nanoparticles, while the appearance of transition metal ionic valence states such as Co^2+^, Co^3+^, and Ni^2+^ may derive from surface oxidation of metallic Co and Ni in air.

**Figure 3:**
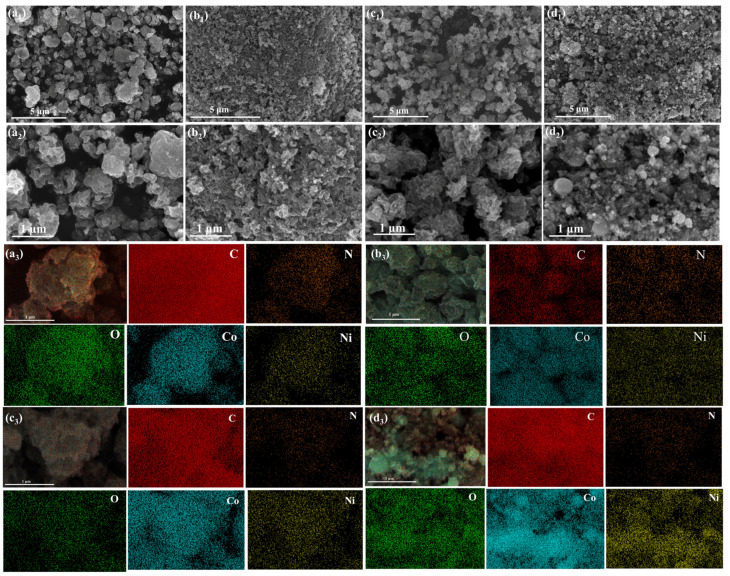
SEM images and EDS mapping of various catalysts: (**a_1_**–**a_3_**) CoNi@NC-550; (**b_1_**–**b_3_**) CoNi@NC-650; (**c_1_**–**c_3_**) CoNi@NC-750; (**d_1_**–**d_3_**) CoNi@NC-850.

The scanning electron microscopy (SEM) showed all samples lost ZIF-67 dodecahedral morphology, and the CoNi@NC-550, CoNi@NC-650, and CoNi@NC-750 samples were fully embedded by one irregular carbon nanocage and CoNi alloy nanoparticles with many carbon nanotubes growing on the surface, and the tips of the tubes were obviously whitish predicted to be CoNi alloy, as shown in Figure 3b_2_. In contrast, the carbon nanocage in the CoNi@NC-850 sample is disrupted and many relatively large CoNi alloy particles can be noticed (Figure 3d_1_,d_2_). The STEM-EDS elemental mapping images show that C, N, O, Co, and Ni are uniformly distributed on the catalyst (Figure 3a_3_–c_3_). Electron layering images show more distribution of Co, Ni in CoNi@NC-850, probably because of the relatively large CoNi alloy particles covering the surface (Figure 3d_3_). According to a growing number of studies, carbon and nitrogen in nitrogen-doped carbon nanotubes form C-N and M-N with transition metals that can enhance catalytic activity [32].

**Figure 4:**
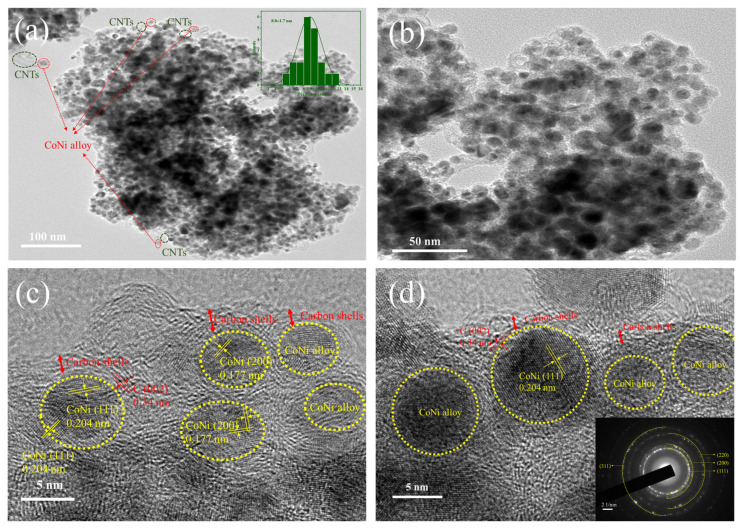
(**a**,**b**) TEM plots of CoNi@NC-650, an inset shows the particle size distribution. (**c**,**d**) HRTEM plots and the corresponding SAED plots of CoNi@NC-650 catalyst.

TEM was used to describe the shape and structure of the CoNi@NC-650 catalyst, and a large number of nanoparticles were evenly dispersed, as seen in Figure 4a. The inset in Figure 4a demonstrates the tiny particle size of the carbon-wrapped alloy nanoparticles, which have an average diameter of 8.8 nm. Part of the CoNi alloy is enclosed by carbon nanotubes at the front section of the tubes, which is due to the catalyst’s catalytic impact of Co and Ni ions on graphitization, as shown in Figure 4b, which is an enlarged TEM picture of a small number of nanoparticles [39]. In Figure 4c,d, HRTEM test results show that the lattice spacings of 0.204 nm and 0.177 nm, which correspond to the (111) and (200) facets of the CoNi alloy, respectively, and that these multi-walled carbon nanotubes are crystalline with a lattice spacing of 0.340 nm, corresponding to the (002) facet of graphitized carbon [40,41]. Additionally, different diffraction crystal surfaces of the CoNi alloy are visible in the matching selected area electron diffraction (SAED) maps, which is compatible with the XRD test findings. TEM and HRTEM for other catalysts are shown in Figure S2 of the Supporting Information. The TEM of CoNi@NC-650 after acid washing is shown in the supporting infographic Figure S3, confirming that the CoNi alloy particles remain in the tube and are not leached out after acid treatment, indicating that the tube is generated by the in situ growth catalyzed by the CoNi metal alloy [42].

**Figure 5:**
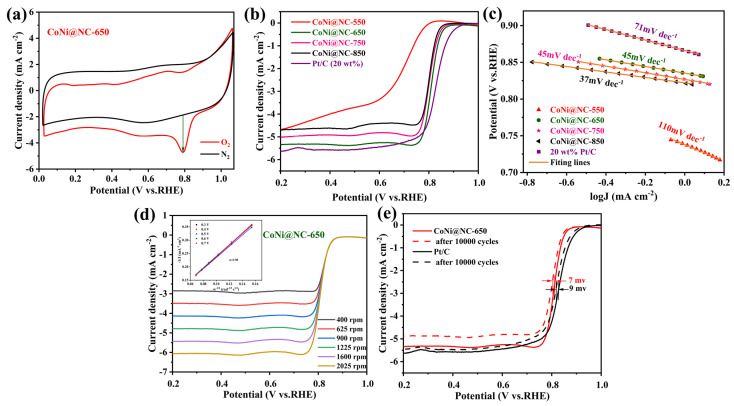
(**a**) CV plots of CoNi@NC-650 under N_2_ and O_2._ (**b**) ORR LSV curves of catalysts. (**c**) Tafel slope (**d**) LSV curves and corresponding K-L plots of CoNi@NC-650 at different rotational speeds. (**e**) LSV curves of CoNi@NC-650 and commercial Pt/C after initial and 10,000 cycles.

To determine the catalytic performance of CoNi@NC-X catalysts, the ORR and OER activities were evaluated in electrolytes of 0.1 M KOH and 1 M KOH, respectively. The activity of the catalysts prepared was first investigated using cyclic voltammetry (CV). As seen in Figure 5a, no significant redox peak was observed in CoNi@NC-650 in an N_2_-saturated at 0.1 M KOH solution, but a distinct cathodic peak can be clearly visible at roughly 0.8 V at oxygen saturation electrolyte, indicating easy oxygen desorption from the surface of the sample for potential outstanding intrinsic electrocatalytic activity toward ORR [43]. The presence of background current was caused by the double-layer charging. Figure 5b compares the polarization curves of CoNi@NC-X catalysts and commercial 20 wt.% Pt/C catalysts for oxygen reduction. The mass activity of commercial Pt/C was calculated to be 0.017 A mg^−1^Pt at 0.9 V vs. RHE and compared commercial Pt/C in other literature in Table S4. It shows that CoNi@NC-650 presents a half-wave potential of 0.81 V, which is higher than that of CoNi@NC-550 (E_1/2_ = 0.68 V), CoNi@NC-750 (E_1/2_ = 0.80 V) and CoNi@NC-850 (E_1/2_ = 0.80 V), and is almost as high as that of Pt/C (E_1/2_ = 0.82 V). In addition, CoNi@NC-650 has an onset potential of 0.89 V at 0.1 mA cm^−2^, close to the onset potential of 0.93 V for Pt/C. The Tafel slope of CoNi@NC-650 is calculated as 45 mv dec^−1^, which is lower than Pt/C (71 mv dec^−1^), indicating that the CoNi@NC has significantly faster ORR kinetics (Figure 5c). To further investigate the catalyst surface redox pathway, the LSV curves of CoNi@NC materials were tested at different potential values (0.30–0.70 V) at 400-2025 rpm, respectively. The electron transfer numbers (n) in the ORR were calculated. The kinetic parameters are analyzed by Koutecky Levich (K-L) equation. Figure 5d shows that the average electron transfer number (n) for the CoNi@NC-650 is 3.98 according to the K-L equation, which is close to the theoretical value of Pt/C of 4. This indicates that the dominant four-electron reaction pathway (O_2_ + 2H_2_O + 4e^−^ = 4OH^−^) in the ORR catalyzed by CoNi@NC under alkaline conditions. The CoNi@NC materials synthesized at other temperatures are also 4 electron transfer as illustrated in the supporting infographic Figure S4. CoNi@NC-650 and commercial Pt/C were also tested with CV cycling between 0.2 and 1.2 V for 10,000 cycles. E_1/2_ at 1600 rpm has only negatively drifted by 7 mV and 9 mV in the LSV curves of CoNi@NC-650 and commercial Pt/C, indicating good ORR stability of the prepared CoNi@NC-650 catalyst.

**Figure 6:**
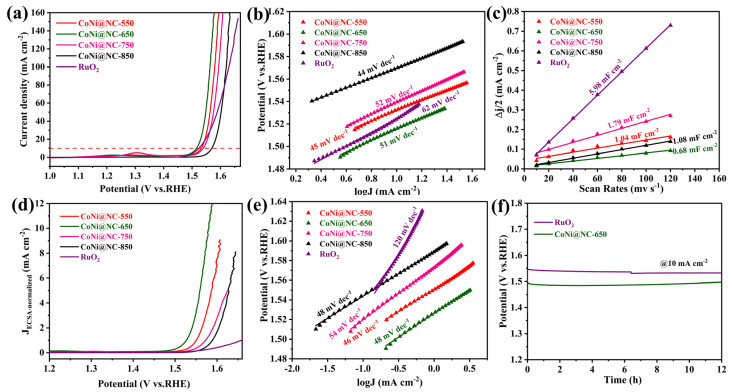
OER activity of CoNi@NC-550, CoNi@NC-650, CoNi@NC-750, CoNi@NC-850, and RuO_2_ catalysts (**a**) LSV curves. (**b**) Tafel slope plots. (**c**) CV current density vs. scan rate (linear slope with respect to bilayer capacitance C_dl_) for different catalysts. (**d**) LSV polarization curves normalized by ECSA for individual catalysts. (**e**) ECSA-normalized Tafel slope. (**f**) Durability tests of CoNi@NC-650 and RuO_2_ at a constant current density of 10 mA cm^−2^.

Figure 6a shows the OER activity of CoNi@NC catalysts produced at various temperatures in 1 M KOH solution. Similarly, the sample obtained at 650 °C exhibited the highest OER performance, with an overpotential of just 286 mV at 10 mA cm^−2^ and 330 mV at 100 mA cm^−2^, both of these are better than the RuO_2_ catalyst (the specific data is shown in Figure S5 of Supporting Information). In addition, as shown in Figure 6b, the Tafel slope of CoNi@NC-650 is 51 mV dec^−1^, is significantly lower than that of RuO_2_ (62 mV dec^−1^), indicating that it is more favorable for the OER reaction kinetics. Figure 6c demonstrates that RuO_2_ has a higher C_dl_ (5.98 mF cm^−2^) than the CoNi@NC-X, which may be due to its tiny nanograin size [44]. It should be noted that the C_dl_ of CoNi@NC-650 is not the highest due to the ECSA of C_dl_ reaction catalyst exposure, whereas the LSV curves usually characterize the corresponding current response at different potentials. Moreover, the high OER activity of CoNi@NC-650 can be due to its high intrinsic activity instead of ECSA, and the optimal OER performance can only be attained by carefully balancing strong intrinsic activity with a large ECSA [45]. LSV curves and Tafel slope plots after ECSA normalized current density are also supplied, as illustrated in Figure 6d,e, to investigate the intrinsic activity of the catalyst (i.e., elimination of ECSA contribution). CoNi@NC-650 still has the lowest overpotential (320 mV at 5 mA cm^−2^) and low Tafel slope (51 mV dec^−1^) after ECSA normalization, indicating that CoNi@NC-650 has the best intrinsic activity. Figure 6f depicts the stability tests of CoNi@NC-650 and RuO_2_ at 10 mA cm^−2^, illustrating that CoNi@NC-650 is extremely stable and the overpotential is consistently lower than RuO_2_ at this current density. These results indicate that CoNi@NC-650 is an effective bifunctional electrocatalyst. Support Information Tables S5 and S6 compare the ORR and OER performance between as-prepared catalysts and also compare them with other bifunctional catalysts in the literature.

We investigate the catalytic active site to gain a better understanding of the catalyst. The remarkable electrocatalytic activity of CoNi@NC is largely due to the structure of the generated NCNT, as well as the overall framework structure. The pyrolysis temperature is essential in the synthesis because the electrocatalytic activity increases and subsequently drops as the temperature rises from 550 to 850 °C. This might be related to the fact that higher pyrolysis temperatures produce lower defect densities, which is due to the higher catalytic activity at these defect sites than at the substrates. In addition to producing catalytically active sites by inducing electrical interactions with adjacent carbon and metal atoms, N-doping also creates structural flaws in the CNT that enable the formation of O_2_ adsorption sites [46,47]. Additionally, the N-doping content of the samples changed with annealing temperatures, where CoNi@NC-650 had the highest nitrogen content (Tables S1 and S7). It was shown that high levels of graphitization and pyridinic-N are both advantageous for ORR reactions and can work together to increase ORR activity. A high level of graphitization considerably reduces the ORR’s electron transfer resistance, whereas pyridinic-N increases defects [48].

**Figure 7:**
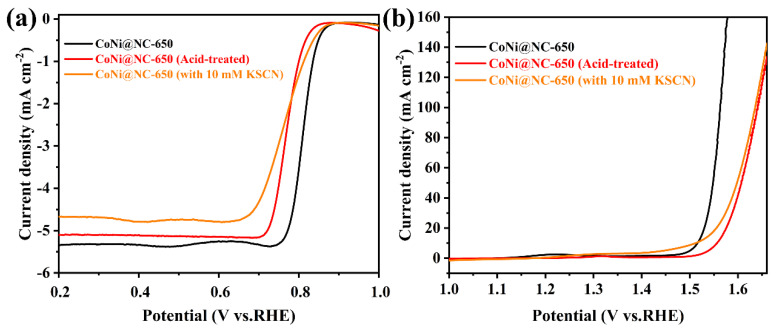
CoNi@NC-650 before and after acid treatment and with 10 mM KSCN (**a**) LSV plots for ORR and (**b**) LSV plots for OER.

Furthermore, some studies and theoretical investigations have demonstrated that the encapsulated CoNi alloys can activate the surrounding graphite layer, which can provide an active site for electrocatalysis [49,50]. We acid-washed the CoNi@NC-650 catalyst with 0.5 M H_2_SO_4_ for 6 h to see if the carbon-coated CoNi metal nanoalloy had any influence on catalytic performance. The EDS energy spectrum content before and after acid-washing shows that the acid treatment etches the metal particles and the CoNi metal is removed in large amounts to reduce the content (Figure S6). Through comparing the ORR and OER from Figure 7a,b, it was found that there is a decrease in both properties after acid washing because of the loss of metal-centered catalytic sites in the material.

In electrocatalysis, SCN^−^ is known to poison the M-Nx active site and is commonly used to detect the presence of M-Nx active sites in catalysts [51]. In this study, 10 mM KSCN was utilized as a probe to investigate whether M-Nx is the catalyst active site in the catalyst. Figure 7a,b indicates that when 10 mM KSCN was added to the electrolyte, the ORR and OER efficiency of something like the catalyst reduced dramatically, indicating that M-Nx is the active material of the CoNi@NC-650 catalyst.

## 4. Conclusions

We report the synthesis of catalysts for nitrogen-doped carbon-encapsulated CoNi bimetallic alloys by pyrolysis of bimetallic organic skeletons under Ar atmosphere. ZIF-67 provides carbon and nitrogen sources for in situ synthesized bimetallic CoNi nanoparticles catalyzed by NCNT growth, urea acts as an inducer and partial nitrogen source for MOF-derived carbon materials, and metals evaporate at high temperatures and are retained in the carbon matrix. The prepared CoNi@NC has excellent ORR/OER bifunctional catalysis and long-term electrochemical stability due to metal-carrier and metal-metal interactions and metal-nitrogen catalysis. We also performed acid washing and SCN^−^ probe assays on the catalysts to demonstrate that the carbon-coated bimetallic nanoparticles and M-Nx are the active substances. The simple method demonstrated within the paper may help in establishing the development of several other MOF-derived carbon material electrocatalysts.

## Data Availability

The data presented in this study are available on request from the corresponding author.

## Supplementary Materials

The following supporting information can be downloaded at: https://www.mdpi.com/article/10.3390/nano13040715/s1, Figure S1: XRD patterns of Co-ZIF, CoNi-ZIF and ZIF-67 simulated; Figure S2: TEM and HRTEM plots of (a1,a2) CoNi@NC-550; (b1,b2) CoNi@NC-750; (c1,c2) CoNi@NC-850; Figure S3: TEM of CoNi@NC-650 (a,b) after acid washing with H_2_SO_4_ for 6 h; Figure S4: LSV curves at different rotating speeds and electron transfer numbers at different potentials of CoNi@NC-550 (a,b), 750 (c,d), 850 (e,f) and commercial Pt/C (g,h) catalysts; Figure S5: Comparison of the overpotential of different catalysts at 10 mA cm^−2^ (η_10_) and 100 mA cm^−2^ (η_100_); Figure S6: EDS of CoNi@NC-650 before (a) and after (b) acid washing reflects change in the content of metal atoms. Table S1: Table of elemental contents of C, N, O, Co and Ni of CoNi@NC catalysts prepared at different temperatures based on XPS analysis; Table S2: Table of the contents of various nitrogen components of CoNi@NC catalysts prepared at different temperatures based on N 1s high-resolution XPS analysis; Table S3: Pore characteristics of CoNi@NC catalysts prepared at different temperatures; Table S4: Comparison of the mass activity of commercial Pt/C with that of commercial Pt/C in other literature; Table S5: Comparison of ORR and OER activity of the as-prepared catalysts; Table S6: Comparison of the ORR and OER catalytic activities of several recently reported MOF-derived non-precious metal carbon-based bifunctional catalysts; Table S7: Table of Atomic content in percent of C, N, O, Co and Ni of CoNi@NC catalysts prepared at different temperatures based on EDS analysis.
