# Supplementary material for: MOF-Derived CoNi Nanoalloy Particles Encapsulated in Nitrogen-Doped Carbon as Superdurable Bifunctional Oxygen Electrocatalyst
Source: Nanomaterials (Basel). 2023 Feb 13;13(4):715. doi: 10.3390/nano13040715 (PMC9961123; doi:10.3390/nano13040715)
Supplement: Supplementary file 1 [file nanomaterials-13-00715-s001.zip › nanomaterials-2119867-supplementary.pdf]

Supplementary Materials

MOF-Derived CoNi Nanoalloy Particles Encapsulated in Nitrogen-Doped Carbon as Superdurable Bifunctional Oxygen Electrocatalyst

Li Wang [1], Jiewen Liu [1], Chuanjin Tian [1,*], Wenyan Zhao [1,*], Pengzhang Li [1], Wen Liu [1], Liang Song [2], Yumin Liu [1], Chang-An Wang [3] and Zhipeng Xie [3]

[1] Institute of New Energy Materials and Devices, School of Materials Science and Engineering, Jingdezhen Ceramic University, Jingdezhen 333001, China
[2] Department of Materials Science and Chemical Engineering, Stony Brook University, Stony Brook, NY 11790, USA
[3] State Key Lab of New Ceramics and Fine Processing, School of Materials Science and Engineering, Tsinghua University, Beijing 100084, China
* Correspondence: tiancj11@139.com (C.T.); zhaowenyan223@126.com (W.Z.)

Experimental section

1. Characterization

Powder X-ray diffraction XRD was obtained on a Rigaku Ultima IV X-ray diffractometer with a scan rate of 1°/2θ min^{-1} using Cu Kα (λ = 0.15406 nm) radiation at 40 kV and 40 mA. Scanning electron microscopy (SEM) images were taken under a Hitachi Regulus 8100 field scanning emission electron microscope (FE-SEM). Transmission electron microscopy (TEM) was taken on a JEM 2100F (200 kV) to analyze the internal structure of the samples. X-ray photoelectron spectroscopy (XPS) was utilized to determine the chemical state and composition of the samples using a Thermo Scientific K-Alpha instrument (USA) and the contaminated carbon peak (284.8 eV) was used as a reference peak to calibrate for the binding energy of other elements. Raman spectra (Raman) were acquired using a 532 nm laser on a Thermo Scientific DXR Raman spectrometer (USA) to analyze the D and G bands. N_2 adsorption-desorption isotherms were obtained on a Quantachrome autosorb iQ instrument (USA), and a liquid nitrogen bath (77 K) and ultra-high purity grade nitrogen and helium were used for nitrogen adsorption experiments to calculate the specific surface area according to the Brunauer-Emmett-Teller (BET) and Barret-Joyer-Halenda (BJH) methods and pore size distribution.

1. Electrode preparation and Electrochemical Measurements

To test the performance of the obtained catalysts, 2.5 mg catalyst was dispersed in a solution of 475 μL ethanol and 25 μL Nafion, sonicated for 20 min to form a homogeneous slurry, and 20 μL catalyst slurry was uniformly dropped on a glassy carbon electrode (GCE) (diameter: 5 mm, area: 0.196 cm^2) as the working electrode. The catalyst loading on the electrodes was 0.51 mg cm^{-2}, compared to 0.1 mg cm^{-2} for commercial Pt/C (20 wt%) and RuO_2. All electrochemical measurements were carried out using a three-electrode system at the CHI660 workstation (Shanghai C&H Instruments Co., Ltd.). The US PIN rotating disc electrode (RDE) was used both for both ORR and OER test at a water bath with a constant temperature of 25°C. All potentials in this study were converted to the reversible hydrogen electrode (RHE). The catalyst-coated GCE and HgO electrodes were used as the working and reference electrode for ORR and OER, while a platinum wire and graphite rod were used as counter electrode for ORR and OER measurement, repectively. N_2 and O_2 were introduced into the solution for 30 min before the ORR electrochemical test. O_2 was introduced for 30 min before the OER test. The ORR (0-1.1 V vs. RHE) and OER (1-2 V vs. RHE) were performed by linear sweep voltammetry (LSV) in 0.1 M KOH and 1 M KOH solutions, respectively, at a sweep rate of 0.01 V/s. The rotating speed of 1600 rpm

was set for OER measurement to avoid the accumulation of oxygen bubbles on the electrode surface. Both ORR and OER curves were iR-corrected. The ORR curves were net of background currents with LSV curves acquired at various rotational speeds of 400, 625, 900, 1225, 1600 and 2025 rpm to calculate the electron transfer numbers of the catalysts.

Table S1. Table of elemental contents of C, N, O, Co and Ni of CoNi@NC catalysts prepared at different temperatures based on XPS analysis.

	Chemical composition (at %)				
	C	N	O	Co	Ni
CoNi@NC-550	77.34	8.17	14.80	1.83	0.85
CoNi@NC-650	73.21	9.70	11.39	3.94	1.77
CoNi@NC-750	81.68	6.59	8.59	1.91	1.23
CoNi@NC-850	85.93	4.22	7.16	1.62	1.08

Table S2. Table of the contents of various nitrogen components of CoNi@NC catalysts prepared at different temperatures based on N 1s high-resolution XPS analysis.

	Nitrogen content (at %)			
	Pyridinic-N	Pyrrolic-N	Graphitic-N	Oxidized-N
CoNi@NC-550	21.58	48.86	14.60	14.95
CoNi@NC-650	20.12	33.96	24.78	24.14
CoNi@NC-750	23.66	26.02	21.88	28.44
CoNi@NC-850	25.36	23.29	39.64	11.70

Table S3. Pore characteristics of CoNi@NC catalysts prepared at different temperatures.

**Sample name**	**BET surface area / $m^2\ g^{-1}$**	**Total pore volume / $cm^3\ g^{-1}$**	**Average pore diameter / nm**
CoNi@NC-550	160	0.21	11.20
CoNi@NC-650	167	0.23	8.59
CoNi@NC-750	119	0.19	9.26
CoNi@NC-850	60	0.12	9.00

Table S4. Comparison of the mass activity of commercial Pt/C with that of commercial Pt/C in other literature.

**Catalyst**	**Test Condition**	**Pt loading ($mg\ cm^{-2}$)**	**Mass Activity ($A\ mg^{-1}Pt$) @0.9V_{RHE}**	**Sweep speed ($mv\ s^{-1}$)**	**Ref.**
Pt/C	0.1 M KOH	0.020	0.017	10	This work
Pt/C	0.1 M $HClO_4$	0.030	0.063	5	[S1]
Pt/C	0.1 M $HClO_4$	0.020	0.110	10	[S2]
Pt/C	0.1 M KOH		0.048	5	[S3]
Pt/C	0.1 M KOH		0.088	5	[S4]
Pt/C	0.1 M KOH	0.030	0.100	10	[S5]
Pt/C	0.1 M KOH		0.038	10	[S6]
Pt/C	0.1 M KOH	0.024	0.068	5	[S7]
Pt/C	0.1 M KOH	0.040	0.023	10	[S8]

Table S5. Comparison of ORR and OER activity of the as-prepared catalysts.

**Catalysts**	**ORR Activity**			**OER Activity**
	**Onset potential**	**Half-wave potential($E_{1/2}$) V (vs. RHE)**	**Limiting current density/$mA\ cm^{-2}$**	**Overpotential@10mA cm^{-2}/mV(η_{10})**

	**V (vs. RHE)**			
CoNi@NC-550	0.79	0.68	4.68	300
CoNi@NC-650	0.89	0.81	5.34	286
CoNi@NC-750	0.87	0.80	5.01	310
CoNi@NC-850	0.85	0.80	4.70	340
Pt/C	0.93	0.82	5.63	N/A
RuO_2	N/A	N/A	N/A	296

Table S6. Comparison of the ORR and OER catalytic activities of several recently reported MOF-derived non-precious metal carbon-based bifunctional catalysts.

**Catalysts**	**ORR Activity**	**OER Activity**	**References**
	**Half-wave potential($E_{1/2}$) V (vs. RHE) 0.1 M KOH**	**Overpotential@10mA cm^{-2}/mV(η_{10}) 1 M KOH**	
**CoNi@NC-650**	0.81	286	This work
MnBDC@75%rGO	0.94	610	[S9]
PANI/ZIF-67	0.75	330	[S10]
FeC@Fe@CNF-2	0.84	440	[S11]
Co_3O_4/HNCP-40	0.834	333	[S12]
Co-MOF@CNTs(5 wt%)	0.82	347	[S13]
FeNiP/NCH	0.75	250	[S14]
Co_9S_8@TDC-900	0.78	330	[S15]
Co@Co3O4@NC-900	0.80	370	[S16]
Co0.25Ni0.75@NCNT30	0.84	410	[S17]
CoNi-MOF/rGO	0.718	318	[S18]
NiFe-LDH/Co,N-CNF	0.79	312 (0.1 M KOH)	[S19]
ZnCo-ZIF@GO	0.76	430 (0.1 M KOH)	[S20]
BSCF@Co-N-C	0.80	400 (0.1 M KOH)	[S21]
CNF@Zn/CoNC	0.82	470 (0.1 M KOH)	[S22]
CoFe/N-HCSs	0.791	290	[S23]
Co_9S_8-MoS_2/N-CNAs@CNFs	0.82	340	[S24]
Co4N@N-CNTs/rGO	0.80	372.1	[S25]
CoNC/NCNTs@CNF	0.78	390	[S26]
3D-CNTA	0.81	360	[S27]

Table S7. Table of Atomic content in percent of C, N, O, Co and Ni of CoNi@NC catalysts prepared at different temperatures based on EDS analysis.

**Sample name**	**Atomic content in percent (at %)**				
	**C**	**N**	**O**	**Co**	**Ni**
CoNi@NC-550	81.05	8.46	7.64	2.34	0.51
CoNi@NC-650	61.01	9.13	7.30	18.14	4.42
CoNi@NC-750	83.25	3.84	3.16	7.88	1.87
CoNi@NC-850	82.35	0.24	5.58	9.62	2.21

Figures

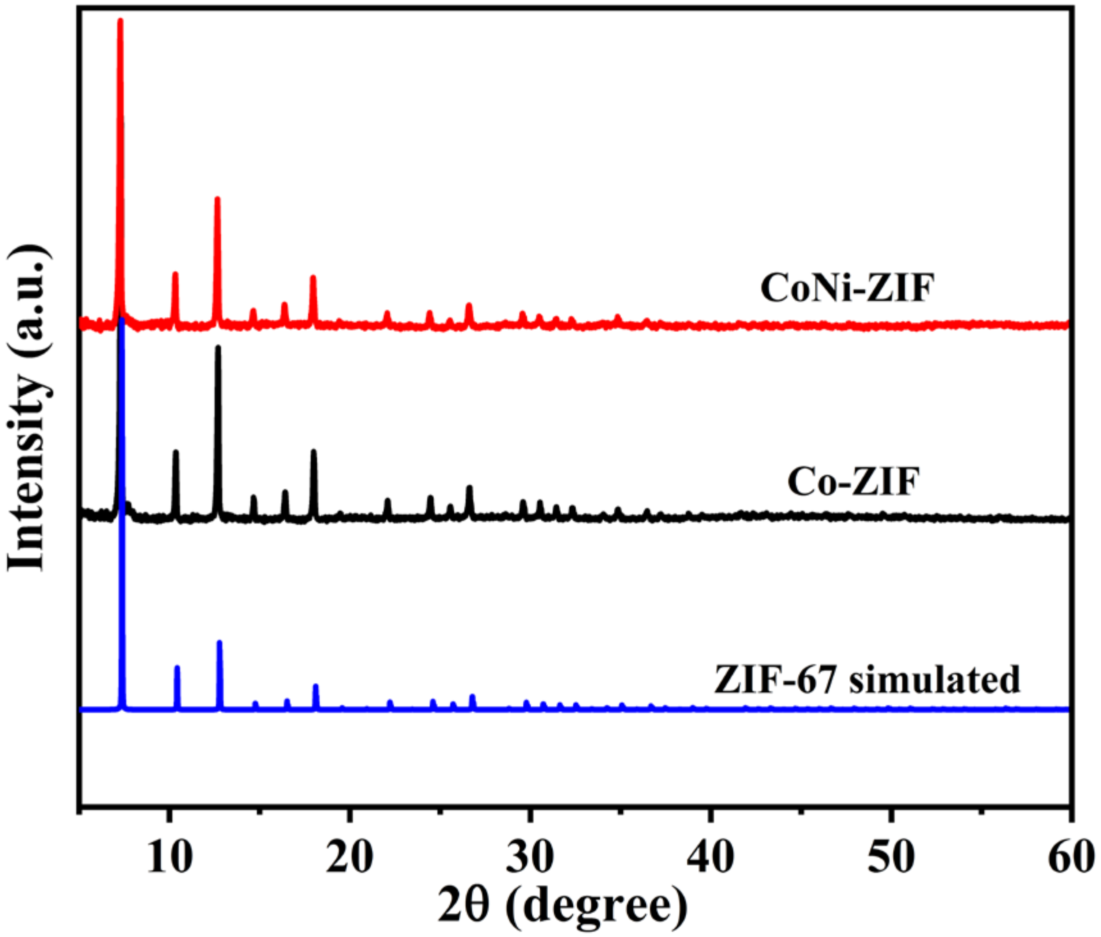

Figure S1. XRD patterns of Co-ZIF, CoNi-ZIF and ZIF-67 simulated.

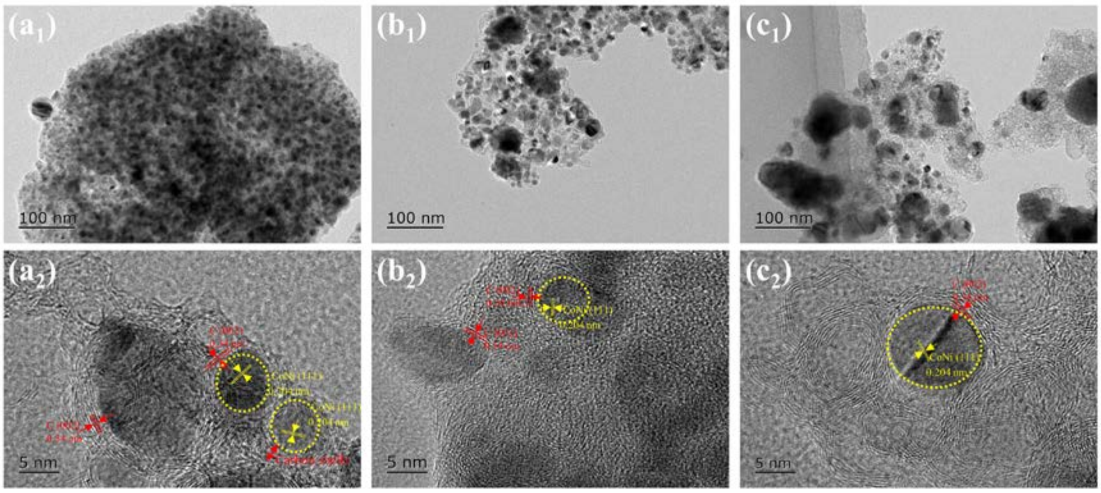

Figure S2. TEM and HRTEM plots of (**a**$_1$–**a**$_2$) CoNi@NC-550; (**b**$_1$–**b**$_2$) CoNi@NC-750; (**c**$_1$–**c**$_2$) CoNi@NC-850.

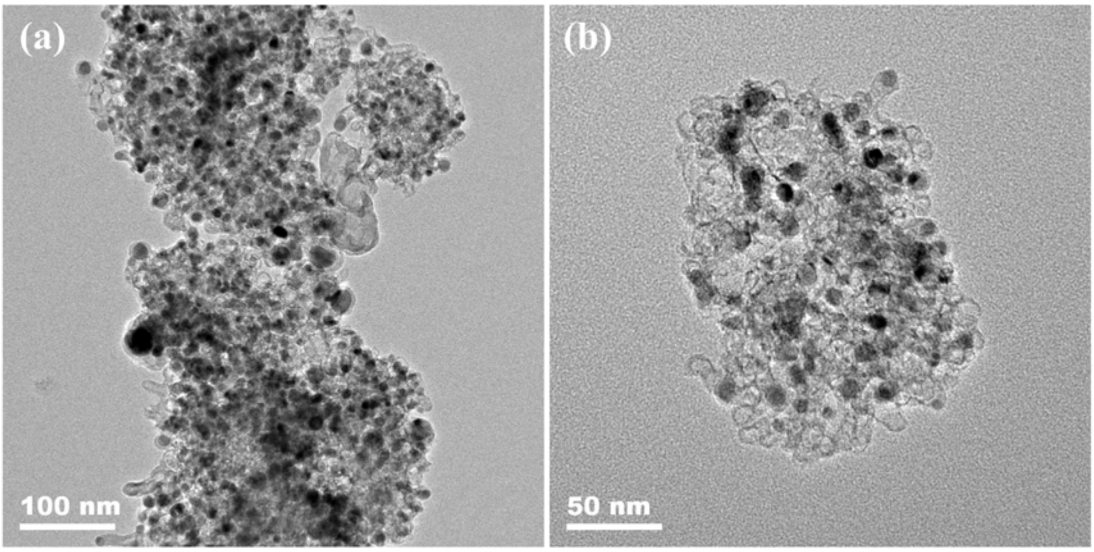

Figure S3. TEM of CoNi@NC-650 (**a–b**) after acid washing with H_2SO_4 for 6h.

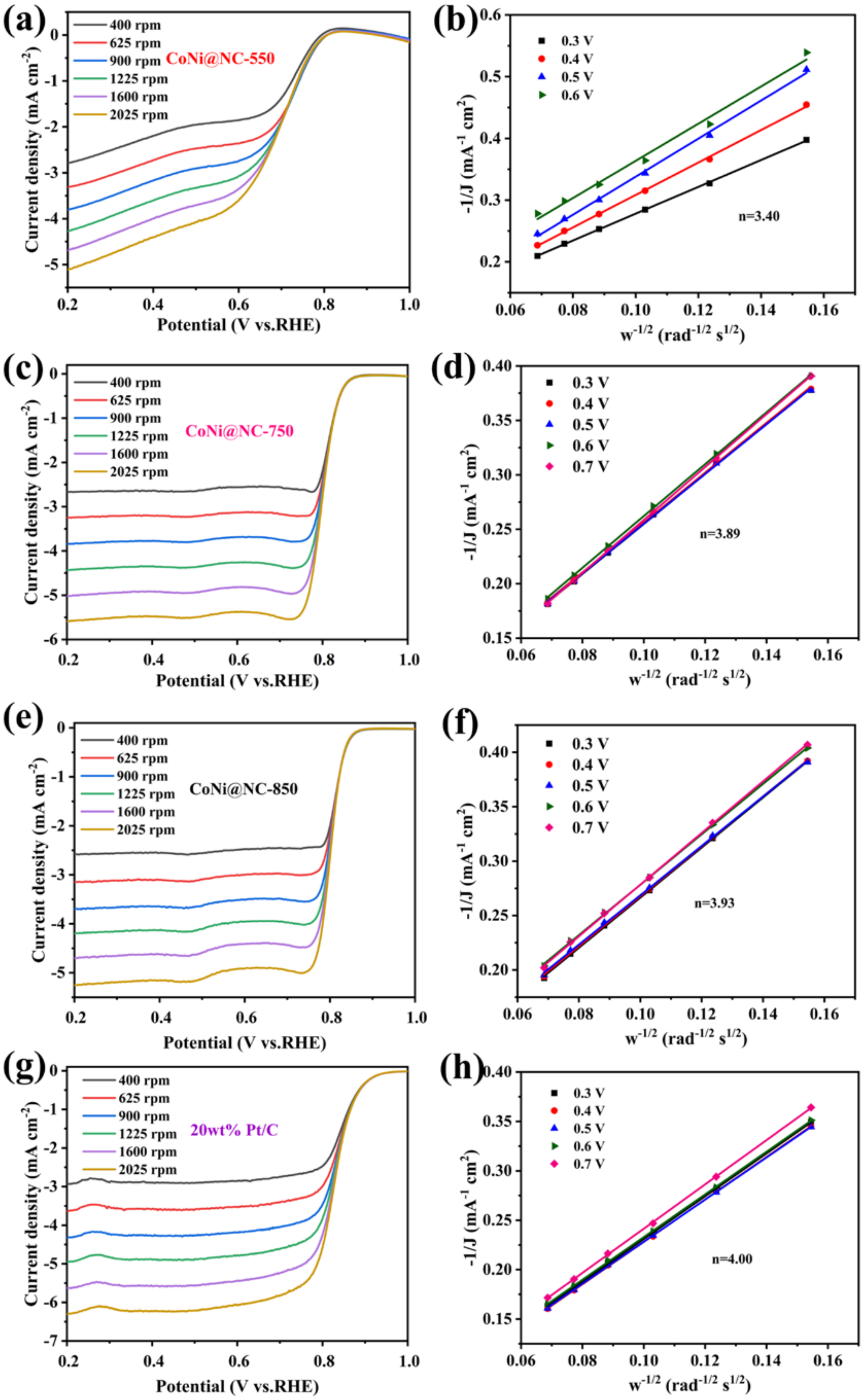

Figure S4. LSV curves at different rotating speeds and electron transfer numbers at different potentials of CoNi@NC-550 (**a**,**b**), 750 (**c**,**d**), 850 (**e**,**f**) and commercial Pt/C (**g**,**h**) catalysts.

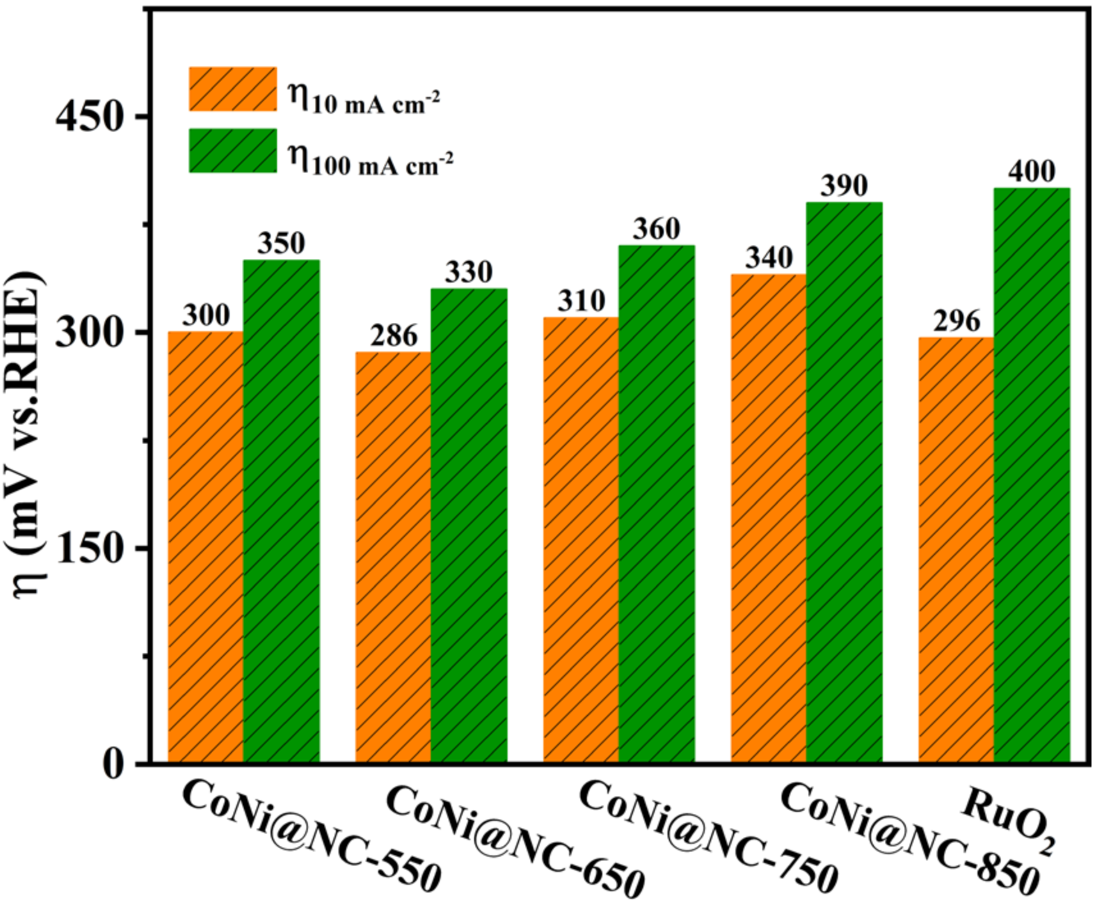

Figure S5. Comparison of the overpotential of different catalysts at 10 mA cm^{-2} (η_{10}) and 100 mA cm^{-2} (η_{100}).

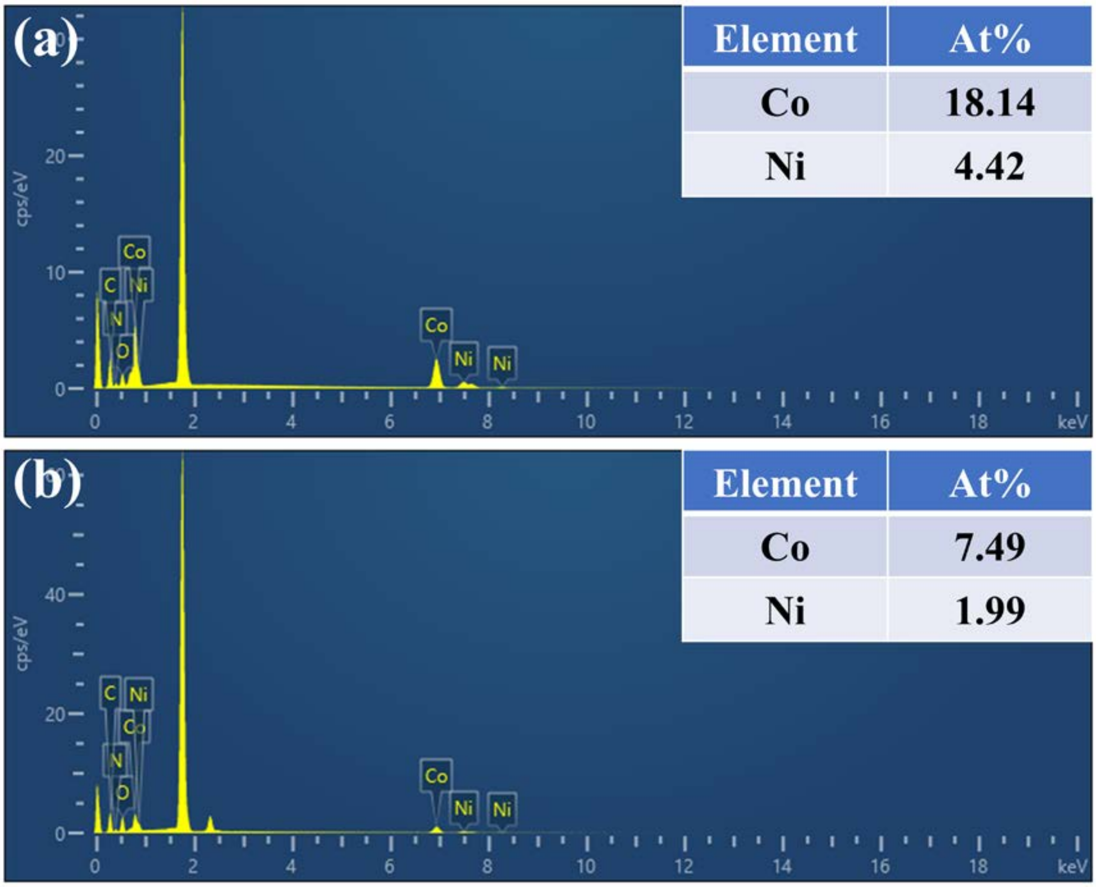

Figure S6. EDS of CoNi@NC-650 before (**a**) and after (**b**) acid washing reflects change in the content of metal atoms.

References

S1. Y. Xiong, Y. Yang, F. J. DiSalvo and H. D. Abruña, "Synergistic bimetallic metallic organic framework-derived Pt–Co oxygen reduction electrocatalysts," *ACS nano*, vol. 14, no. 10, pp. 13069-13080, 2020.

S2. J. Liang, N. Li, Z. Zhao, L. Ma, X. Wang, S. Li, X. Liu, T. Wang, Y. Du, G. Lu, J. Han, Y. Huang, D. Su and Q. Li, "Tungsten-Doped L1(0) -PtCo Ultrasmall Nanoparticles as a High-Performance Fuel Cell Cathode," *Angew Chem Int Ed Engl*, vol. 58, no. 43, pp. 15471-15477, 2019.

S3. W. Xiao, M. A. L. Cordeiro, M. Gong, L. Han, J. Wang, C. Bian, J. Zhu, H. L. Xin and D. Wang, "Optimizing the ORR activity of Pd based nanocatalysts by tuning their strain and particle size," *Journal of Materials Chemistry A*, vol. 5, no. 20, pp. 9867-9872, 2017.

S4. G. Wang, J. Guan, L. Xiao, B. Huang, N. Wu, J. Lu and L. Zhuang, "Pd skin on AuCu intermetallic nanoparticles: A highly active electrocatalyst for oxygen reduction reaction in alkaline media," *Nano Energy*, vol. 29, pp. 268-274, 2016.

S5. K. Qi, W. Zheng and X. Cui, "Supersaturation-controlled surface structure evolution of Pd@Pt core-shell nanocrystals: enhancement of the ORR activity at a sub-10 nm scale," *Nanoscale*, vol. 8, no. 3, pp. 1698-1703, 2016.

S6. S. Hussain, H. Erikson, N. Kongi, M. Merisalu, P. Ritslaid, V. Sammelselg and K. Tammeveski, "Heat-treatment effects on the ORR activity of Pt nanoparticles deposited on multi-walled carbon nanotubes using magnetron sputtering technique," *International Journal of Hydrogen Energy*, vol. 42, no. 9, pp. 5958-5970, 2017.

S7. Z. Cui, G. Fu, Y. Li and J. B. Goodenough, "Ni(3) FeN-Supported Fe(3) Pt Intermetallic Nanoalloy as a High-Performance Bifunctional Catalyst for Metal-Air Batteries," *Angew Chem Int Ed Engl*, vol. 56, no. 33, pp. 9901-9905, 2017.

S8. D. Chen, Z. Li, Y. Zhou, X. Ma, H. Lin, W. Ying and X. Peng, "Fe(3)Pt intermetallic nanoparticles anchored on N-doped mesoporous carbon for the highly efficient oxygen reduction reaction," *Chem Commun (Camb)*, vol. 56, no. 36, pp. 4898-4901, 2020.

S9. A. Wahab, N. Iqbal, T. Noor, S. Ashraf, M. A. Raza, A. Ahmad and U. A. Khan, "Thermally reduced mesoporous manganese MOF@ reduced graphene oxide nanocomposite as bifunctional electrocatalyst for oxygen reduction and evolution," *RSC advances*, vol. 10, no. 46, pp. 27728-27742, 2020.

S10. M. Khalid, A. M. B. Honorato, H. Varela and L. Dai, "Multifunctional electrocatalysts derived from conducting polymer and metal organic framework complexes," *Nano Energy*, vol. 45, pp. 127-135, 2018.

S11. J. T. Liu, Y. Xie, Q. Gao, F. H. Cao, L. Qin, Z. Y. Wu, W. Zhang, H. Li and C. L. Zhang, "1D MOF-Derived N-Doped Porous Carbon Nanofibers Encapsulated with Fe3C Nanoparticles for Efficient Bifunctional Electrocatalysis," *European Journal of Inorganic Chemistry*, vol. 2020, no. 6, pp. 581-589, 2020.

S12. D. Ding, K. Shen, X. Chen, H. Chen, J. Chen, T. Fan, R. Wu and Y. Li, "Multi-level architecture optimization of MOF-templated Co-based nanoparticles embedded in hollow N-doped carbon polyhedra for efficient OER and ORR," *Acs Catalysis*, vol. 8, no. 9, pp. 7879-7888, 2018.

S13. Y. Fang, X. Li, F. Li, X. Lin, M. Tian, X. Long, X. An, Y. Fu, J. Jin and J. Ma, "Self-assembly of cobalt-centered metal organic framework and multiwalled carbon nanotubes hybrids as a highly active and corrosion-resistant bifunctional oxygen catalyst," *Journal of Power Sources*, vol. 326, pp. 50-59, 2016.

S14. Y.-S. Wei, M. Zhang, M. Kitta, Z. Liu, S. Horike and Q. Xu, "A Single-Crystal Open-Capsule Metal–Organic Framework," *Journal of the American Chemical Society*, vol. 141, no. 19, pp. 7906-7916, 2019.

S15. J.-Y. Zhao, R. Wang, S. Wang, Y.-R. Lv, H. Xu and S.-Q. Zang, "Metal–organic framework-derived Co9S8 embedded in N, O and S-tridoped carbon nanomaterials as an efficient oxygen bifunctional electrocatalyst," *Journal of Materials Chemistry A*, vol. 7, no. 13, pp. 7389-7395, 2019.

S16. Z. Guo, F. Wang, Y. Xia, J. Li, A. G. Tamirat, Y. Liu, L. Wang, Y. Wang and Y. Xia, "In situ encapsulation of core–shell-structured Co@Co3O4 into nitrogen-doped carbon polyhedra as a bifunctional catalyst for rechargeable Zn–air batteries," *Journal of Materials Chemistry A*, vol. 6, no. 4, pp. 1443-1453, 2018.

S17. A. Kundu, A. Samanta and C. R. Raj, "Hierarchical Hollow MOF-Derived Bamboo-like N-doped Carbon Nanotube-Encapsulated Co(0.25)Ni(0.75) Alloy: An Efficient Bifunctional Oxygen Electrocatalyst for Zinc-Air Battery," *ACS Appl Mater Interfaces*, vol. 13, no. 26, pp. 30486-30496, 2021.

S18. X. Zheng, Y. Cao, D. Liu, M. Cai, J. Ding, X. Liu, J. Wang, W. Hu and C. Zhong, "Bimetallic Metal-Organic-Framework/Reduced Graphene Oxide Composites as Bifunctional Electrocatalysts for Rechargeable Zn-Air Batteries," *ACS Appl Mater Interfaces*, vol. 11, no. 17, pp. 15662-15669, 2019.

S19. Q. Wang, L. Shang, R. Shi, X. Zhang, Y. Zhao, G. I. N. Waterhouse, L.-Z. Wu, C.-H. Tung and T. Zhang, "NiFe Layered Double Hydroxide Nanoparticles on Co, N-Codoped Carbon Nanoframes as Efficient Bifunctional Catalysts for Rechargeable Zinc-Air Batteries," *Advanced Energy Materials*, vol. 7, no. 21, 2017.

S20. Y. Xiao, B. Guo, J. Zhang, C. Hu, R. Ma, D. Wang and J. Wang, "A bimetallic MOF@graphene oxide composite as an efficient bifunctional oxygen electrocatalyst for rechargeable Zn-air batteries," *Dalton Trans*, vol. 49, no. 17, pp. 5730-5735, 2020.

S21. Y. Arafat, M. R. Azhar, Y. Zhong, X. Xu, M. O. Tade and Z. Shao, "A Porous Nano-Micro-Composite as a High-Performance Bi-Functional Air Electrode with Remarkable Stability for Rechargeable Zinc-Air Batteries," *Nanomicro Lett*, vol. 12, no. 1, pp. 130, 2020.

S22. Y. Zhao, Q. Lai, J. Zhu, J. Zhong, Z. Tang, Y. Luo and Y. Liang, "Controllable Construction of Core-Shell Polymer@Zeolitic Imidazolate Frameworks Fiber Derived Heteroatom-Doped Carbon Nanofiber Network for Efficient Oxygen Electrocatalysis," *Small*, vol. 14, no. 19, pp. e1704207, 2018.

S23. J. Li, Y. Kang, W. Wei, X. Li, Z. Lei and P. Liu, "Well-dispersed ultrafine CoFe nanoalloy decorated N-doped hollow carbon microspheres for rechargeable/flexible Zn-air batteries," *Chemical Engineering Journal*, vol. 407, 2021.

S24. W. Zhang, X. Zhao, Y. Zhao, J. Zhang, X. Li, L. Fang and L. Li, "Mo-Doped Zn, Co Zeolitic Imidazolate Framework-Derived Co(9)S(8) Quantum Dots and MoS(2) Embedded in Three-Dimensional Nitrogen-Doped Carbon Nanoflake Arrays as an Efficient Trifunctional Electrocatalysts for the Oxygen Reduction Reaction, Oxygen Evolution Reaction, and Hydrogen Evolution Reaction," *ACS Appl Mater Interfaces*, vol. 12, no. 9, pp. 10280-10290, 2020.

S25. H. Qi, Y. Feng, Z. Chi, Y. Cui, M. Wang, J. Liu, Z. Guo, L. Wang and S. Feng, "In situ encapsulation of Co-based nanoparticles into nitrogen-doped carbon nanotubes-modified reduced graphene oxide as an air cathode for high-performance Zn-air batteries," *Nanoscale*, vol. 11, no. 45, pp. 21943-21952, 2019.

S26. X. Yao, J. Li, Y. Zhu, L. Li and W. Zhang, "Designed synthesis of three-dimensional callistemon-like networks structural multifunctional electrocatalyst: Graphitic-carbon-encapsulated Co nanoparticles/N-doped carbon nanotubes@carbon nanofibers for Zn-air batteries application," *Composites Part B: Engineering*, vol. 193, 2020.

S27. S. Wang, J. Qin, T. Meng and M. Cao, "Metal–organic framework-induced construction of actiniae-like carbon nanotube assembly as advanced multifunctional electrocatalysts for overall water splitting and Zn-air batteries," *Nano Energy*, vol. 39, pp. 626-638, 2017.
